# Nervous Patients

**Published:** 1899-08

**Authors:** Walter F. Lewis


ARTICLE V.

### NERVOUS PATIENTS.

DR. WALTER F. LEWIS.

We speak of a person as being nervous, or in a condition of nervousness, as indicating an abnormal condition of the nervous system, while it may mean a condition of health as well as disease, as, for example, we speak of a "man of nerve," "a strong nervous man," or a "weak nervous woman," according to the different acceptation of the word nervous. The nerves are so intimately connected with the whole body that we ought to find healthy and vigorous nervous conditions in a healthy body, and vice versa, and this in a large measure is doubtless true; but there may exist an hereditary predisposition which interferes with the normal functions of the nervous system in an apparently healthy body, thereby causing us to estimate greater fortitude and power of endurance than the nerves will sustain. With the ancients the nerves were so intimately associated with the tendons and ligaments that nervousness indicated strength, etc.

Without, therefore, going into any homily upon the nervous system, I desire to make some suggestion that may be of value to us in our contact with that class of people whom I have pleased to denominate "nervous patients," those who suffer the most acutely, and make operations in the oral cavity more difficult than any other class. The close relationship of the brain with the nervous system makes the dread of the operation almost as terrible as the operation itself. It goes without saying that the average patient dreads the dental chair. "I hate it, I hate it, and who shall dare to chide me for hating the dental chair" is a sentiment which finds lodgment in the breast of all "nervous patients." The dread of suffering is greatly intensified by this condition, and suffering to such patients is one of the greatest factors in human experience. It enters into every avenue of life. It stands like a grim specter in every household. It looks upon every scene of joy and mirth. It enters the palace and hovel alike. The heart that throbs beneath ermine and velvet dreads it as well as that of the savage in his tepee. It comes unannounced in the darkest midnight hour, and likewise under the blaze of the noonday sun. It is no respector of persons, breathing its blighting poison upon the helpless infant, as well as on the vigorous and strong. It lurks beneath the smile of the beauty of the drawing room, and shows its masked face behind the fervid utterances of the orator. It is not limited to space, but pursues its victim with relentless energy to the highest mountain peak or the lowliest vale, and it is this influence which causes our nervous patient to dread dental operations. The question naturally arises, "What are we going to do with these conditions which are not mere idiosyncrasies, but are conditions over which the patient can have no possible control?" We offer some suggestions which may or may not meet with your approval, but if they are instrumental in causing you to give greater consideration to this subject, the object of the paper will have been accomplished. It is of paramount importance, when these patients present themselves for operations, to study carefully their peculiarities of temperament, habits of thought, etc. If necessary, humor any or all of their harmless whims, agreeing with them in so far as is consistent with true professional dignity, and above all strive to gain their confidence, without which it is hardly possible to operate in a satisfactory manner. Avoid all display of instruments or appliances which have a tendency to excite apprehension that pain lurks behind their use. Cultivate a bright and cheerful manner without descending to frivolity or buffoonery. Do whatever is required in prompt and reliant manner without any unnecessary motion or display. Make the sitting of such length as is compatible with the ability of the patient to bear the nervous strain of the operation. Never, under any circumstances, go beyond this limit. There are many operators who excel in manipulative ability, but who are totally devoid of a proper discrimination as to the patient's power of endurance. How often we have heard the remark, "I sat in the chair three, four or five hours," or, possibly, "all day;" "had a dozen or more cavities filled, and was utterly used up for the week following." Such lack of judgment and proper discernment are on the part of an operator for a nervous patient's strength only commensurate with a blacksmith nailing the shoes upon the hoofs of a horse. In many cases these patients are suffering under an extreme nervous tension from the moment they are seated in the chair until the sitting is finished. Nay, more than that, the dread which has been constantly in their minds from the time the appointment was made until its fulfillment is a veritable nightmare filled with apparitions of dental engines, "dam clamps," hot air syringes, nerve broaches, mallets, etc., so that they commence the sitting in a state of nervous suspense bordering on frenzy. With such patients half an hour or at most an hour is quite a sufficient tax upon their nerve capital, and should not be repeated oftener than once a week at most, unless the case is of unusual urgency, and even then it is of doubtful expediency. In case of a threatened nervous collapse it is better to dismiss your patient even though you have not accomplished much. The next sitting will undoubtedly be more fruitful of results, besides convincing your patient that your interest in their welfare is not altogether measured by dollars and cents. Don't be afraid to exhibit proper sympathy unembellished with gush. Never say in a frigid tone that would "freeze up the pump," or "strike terror to the heart of a grtndstone," "You must expect it to hurt; you can't expect to have teeth filled without pain." You might as well hit them over the head with an Indian war club, and then wonder that they shrink from your touch and manner. Be as gentle in your manner and touch as is consistent with thoroughness and quality of your operation. Do not become imbued with the idea that it is ever necessary io resort to harsh or heroic measures with such patients, either in your mental attitude or in the demands of the operation. You would better fill a tooth with gutta-percha or some material easily inserted than with a mistaken conviction of excellence set a bur tearing into sensitive tissues (for the sake of undercuts for a gold plug) that thrills and throbs through the whole nervous economy like an electric shock. While it is true that gold is the best material with which to fill teeth, there may be circumstances where its introduction could only be accomplished with dire results to your patient's nervous economy. The permanence of the operation is not so important as the conservation of your patient's nervous strength. The necessity for inflicting pain must not be measured by the seeming ability to bear it. A shock given the nerve centers may produce results more inimical to the health of your patient than the presence of a dozen carious teeth.

While I am not prepared to indorse hypnotism in the broadest acceptance of the term, I nevertheless believe that much may be done to alleviate the fear of the nervous patient by directing his mind into channels entirely remote from the one which fill his whole being with a nameless terror, viz., the dread of the operation. If this should necessitate some outlay of mental effort upon your part, surely the end ought to justify the means. It is hardly necessary to direct your attention to the matter of externals in the way of appointment. That, of course, will be one of your fundamental requisites. An object may be of such a character as to catch the eye, and through that influence direct the thought away from unpleasant association, thus, in a measure at least, neutralizing the emotion caused by fear of pain. A picture suggestive of the beauties of nature—singing birds and blooming flowers—would be somewhat more appropriate than, for example, Pocahontas saving the life of Captain John Smith, or some equally pleasant theme.

The use of tonic, stimulants or sedatives in certain nervous conditions is not only permissible but actually desirable, especially when it is necessary to carry the patient through an ordeal of pain such as the excavation of a hyper-sensitive tooth. Of course, their use should always be accompanied with intelligent and proper discrimination. It is well in some cases to prepare the patient for the sitting by the use of tonics administered in advance. There are many which will prove effectual in such cases; it is not necessary to specify any particular one, and as all operations in the oral cavity are in more or less degree surgical, it will be found desirable to emyloy opiates or anesthetics when circumstances seem to indicate their use.

My own experience leads me to believe that inhalation is attended with less evil effects than using the stomach as a vehicle to carry the drug into the circulation, as this method will prove a greater tax upon the system, while the effects will be of longer duration than is necessary. While many operators use morphine, Jamaica dogwood and other agents, my experience has been more satisfactory with chloroform, ether or nitrous oxide gas. Their use should be attended with great care, and only small quantities administered, especially chloroform, with which the results can often be attained by a few drops quickly inhaled, rendering the excavation of very sensitive dentine comparatively painless. In the use of all these agents, kind, quality and quantity must be regulated by the knowledge and juagment of the operator. Plenty of fresh, pure air, with abundance of sunshine, will go far towards dispelling fears and banishing the hobgoblins of dread in our nervous patients.

It would be difficult to outline a specific formula for all cases; there will be found peculiar phases of temperament in each case which claims our service. Consideration coupled with tact and skill will enable us to smooth many of the rough places in the path of this class of patients.—*Pacific Medico-Dental Gazette.*

